# Cryptic Species in Putative Ancient Asexual Darwinulids (Crustacea, Ostracoda)

**DOI:** 10.1371/journal.pone.0039844

**Published:** 2012-07-03

**Authors:** Isa Schön, Ricardo L. Pinto, Stuart Halse, Alison J. Smith, Koen Martens, C. William Birky

**Affiliations:** 1 Freshwater Biology, Royal Belgian Institute of Natural Sciences, Brussels, Belgium; 2 Department of Biology, University of Hasselt, Diepenbeek, Belgium; 3 Institute of Geosciences, University of Brasília, Brasilia, Brazil; 4 Bennelongia Pty Ltd, Wembley, Austrália; 5 Department of Geology, Kent State University, Kent, Ohio, United States of America; 6 Department of Biology, University of Ghent, Gent, Belgium; 7 Department of Ecology and Evolutionary Biology, University of Arizona, Tucson, Arizona, United States of America; Natural History Museum of Denmark, University of Copenhagen, Denmark

## Abstract

**Background:**

Fully asexually reproducing taxa lack outcrossing. Hence, the classic Biological Species Concept cannot be applied.

**Methodology/Principal Findings:**

We used DNA sequences from the mitochondrial COI gene and the nuclear ITS2 region to check species boundaries according to the evolutionary genetic (EG) species concept in five morphospecies in the putative ancient asexual ostracod genera, *Penthesilenula* and *Darwinula*, from different continents. We applied two methods for detecting cryptic species, namely the K/θ method and the General Mixed Yule Coalescent model (GMYC). We could confirm the existence of species in all five darwinulid morphospecies and additional cryptic diversity in three morphospecies, namely in *Penthesilenula brasiliensis, Darwinula stevensoni* and in *P. aotearoa.* The number of cryptic species within one morphospecies varied between seven (*P. brasiliensis)*, five to six (*D. stevensoni*) and two (*P. aotearoa*), respectively, depending on the method used. Cryptic species mainly followed continental distributions. We also found evidence for coexistence at the local scale for Brazilian cryptic species of *P. brasiliensis* and *P. aotearoa*. Our ITS2 data confirmed that species exist in darwinulids but detected far less EG species, namely two to three cryptic species in *P. brasiliensis* and no cryptic species at all in the other darwinulid morphospecies.

**Conclusions/Significance:**

Our results clearly demonstrate that both species and cryptic diversity can be recognized in putative ancient asexual ostracods using the EG species concept, and that COI data are more suitable than ITS2 for this purpose. The discovery of up to eight cryptic species within a single morphospecies will significantly increase estimates of biodiversity in this asexual ostracod group. Which factors, other than long-term geographic isolation, are important for speciation processes in these ancient asexuals remains to be investigated.

## Introduction

The taxonomy and nomenclature of organisms reproducing without meiosis in a non-Mendelian way (clonal, vegetative, asexual, etc.) are long-standing problems, which have often generated heated debates and for which various (and sometimes conflicting) solutions have been proposed (reviews in [1], [2], [3], [4]). Mayr [5] admitted to resorting to the morphological species concept when dealing with clonal organisms, but treated the problem as marginal. Despite a tendency to regard asexuals as evolutionary dead ends [6] (but see [7], [8]), asexuality is not at all rare in animals [9] and, for numerous pro- and eukaryotes, asexuality is rather the rule than the exception. Furthermore, there are a number of eukaryote groups which seem to have reproduced without any sex for millions of years, the so-called ancient asexual scandals [10]. To date, four putative ancient asexual animal groups are known: bdelloid rotifers [11], some lineages of oribatid mites [12] and stick insects [13], and darwinulid ostracods [14], [15].

Martens et al. [3] argued that the key to taxonomy of organisms reproducing clonally is in the distinction between different kinds of clones, their different origins and their different modes of reproduction. Barraclough et al. [16] used population genetic theory to show that asexual organisms can, in principle, undergo speciation, either as a result of diversifying selection for adaptation to different niches or by long-term physical isolation. Birky [17] and [18] suggested that the theory of [16] was a version of the evolutionary species concept that was grounded in evolutionary and population genetic theory and could be called the evolutionary genetic (EG) species concept. To assign asexual organisms to evolutionary genetic species, Birky [19] devised a species criterion originally called the 4× rule, now called the K/θ method. It identifies species as samples from populations which have been evolving independently long enough to form clusters (clades) separated by gaps too deep to be ascribed to random genetic drift within a species; such gaps can only be formed by diversifying selection or by long-term physical isolation (for example, by separate evolution on different continents). This represents speciation. Gaps due to drift alone have an average depth of 2 N_e_ generations, with a 95% confidence interval of 4 N_e_ generations. Thus, species can be identified as sister clades separated by gaps greater than t = 4 N_e_ generations deep. At that point the sequence difference between the clusters will be K = 8 N_e_μ where μ is the mutation rate per site, while the mean sequence difference within a cluster will be θ ≈ 2 N_e_μ; thus K/θ >8 N_e_μ/2 N_e_μ = 4.

Subsequently, several authors [20]–[25] applied a different species criterion devised by [26] called the General Mixed Yule Coalescent model (GMYC). It uses a likelihood procedure to identify the point at which the rate of branching in a phylogenetic tree increases dramatically as it goes from speciation processes to branching of lineages within populations. The GMYC model distinguishes different species from subpopulations within a species, because it will not identify populations as independently evolving if they exchange more than about one individual per thousand generations [27]. The application of the K/θ method is independent of population structure, which is accounted for in the effective population size that cancels out in the ratio K/θ.

Both methods were applied to datasets from bdelloid rotifers and oribatid mites [17], [18] and to ostracods [28] and other invertebrates, fungi and protists [17]; in general, both methods identify the same (or similar) entities. Finding independently evolving populations using two different tests is strong evidence that processes akin to speciation occur in asexual organisms and that the identified entities are similar, if not identical, to classic species. As Coyne [29] pointed out, the fact that ancient asexuals are organized in discrete units, like sexual species, contributes towards our understanding of why organic forms in general are discontinuous.

Bdelloid rotifers comprise more than 460 classic species [30]. Within oribatid mites, the Desmonomata have approximately 400 parthenogenetic species and two families from the Enarthronota hold another 330 parthenogenetic species [31] (but see [32]). Additionally, more than 30 cryptic species have been reported in certain bdelloids [23], [25]. In contrast to this speciosity, the ostracod family Darwinulidae holds about 30 classic (morphology-based) species in 6 genera [15], [33], [34]. Even with newly described species (see, e.g. [34]–[41]) and species as yet undescribed, the overall number of recent darwinulid morphospecies will most likely not exceed 50, this despite taxonomic efforts comparable to those for bdelloid rotifers. Darwinulids thus offer an interesting comparative opportunity to apply the EG species concept to improve our understanding of evolutionary processes in putative ancient asexual animal taxa, in order to test whether speciation processes in the Darwinulidae differ from those of bdelloid rotifers and oribatid mites, as they apparently have resulted in a much lower overall number of species.

The phylogeny of the Darwinulidae has been investigated with morphological [34], [42] and molecular data [42], [43]. With one exception [34], morphological intermediates between genera within the family are lacking [44]. At lower taxonomic levels, however, molecular and morphological data are so far available for one species only, namely *Darwinula stevensoni* Brady and Robertson, 1870 [45]–[48].

In contrast to *Darwinula* which has now only one recent phenotypic species (although there could have been more fossil species in the past), *Penthesilenula* is one of the most speciose genera within the Darwinulidae with currently 11 morphospecies. Pinto et al. [37] encountered some difficulties when applying the classic phenotypic species concept to Brazilian *Penthesilenula* species. Soft part morphologies (mainly chaetotaxy of limbs) were almost identical, while it remained unclear whether the observed differences in valve shape in *P. brasiliensis* have a genetic or environmental background. This makes the genus *Penthesilenula* into an ideal test case for the application of the K/θ and GMYC methods to detect cryptic evolutionary genetic species in darwinulid ostracods.

We use both mitochondrial COI and nuclear ITS2 sequence data for our analyses. Although the evolution of mitochondrial and nuclear genomes is expected to be linked in ancient asexuals, earlier research has indicated differences between these genomes in *Darwinula stevensoni* [46], with no genetic variability at all in ITS1, but some in COI. This is explained by different rates of molecular evolution [43] as well as homogenizing mechanisms in the ribosomal part of the nuclear genome of darwinulids, counteracting the expected accumulation of mutations [47].

Here, we apply the EG species concept to the genera *Penthesilenula* and *Darwinula* with a four-fold aim: first, we test whether species can be found in two well-defined genera of darwinulid ostracods; second, we test whether variation in nuclear or mitochondrial sequence data is sufficient and which of the two is more suitable for identifying species boundaries in long-term asexuals; third, we compare the results of species and speciation processes between a species-rich and a species-poor ostracod genus of similar fossil age; and fourth, we compare our results with those on bdelloid rotifers and oribatid mites to draw more general conclusions on the processes potentially leading to speciation in putative ancient asexuals.

## Results

### COI

According to jModeltest [49] the best evolutionary model for the entire data set (with the outgroup) was Tamura-Nei+ Invariable sites + Gamma distribution (TrN+I+G) with the following parameters: freqA = 0.34, freqC = 0.26, freqG = 0.11 and freqT = 0.28. Rate matrices varied between 1.00 and 10.87 (R(a) [A–C] = 1.00; R(b) [A–G] = 4.30; R(c) [A–T] = 1.00; R(d) [C–G] = 1.00; R(e) [C–T] = 10.87; R(f) [G–T] = 1.00). The proportion of invariable sites was 0.58 and the gamma distribution shape parameter equaled 1.57. Likelihoods of the Maximum-Likelihood (ML) trees constructed from COI data with and without clock assumption in PAUP [50] did not differ significantly from each other (−2ΔLL = 55.62, df = 52, p>0.10), indicating that our assumption of equal molecular rates was probably appropriate (but see [51]).

### Phylogenetic Relationships

**Figure 1:**
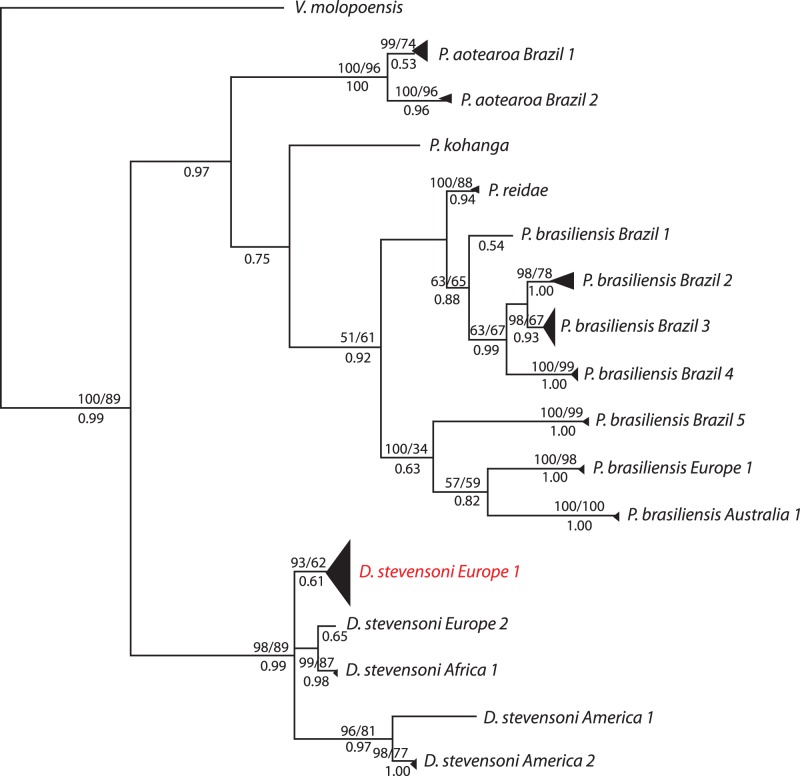
COI phylogeny of the three darwinulid genera. This phylogeny was obtained by Bayesian Inference. Height of triangles reflects the number of sequences. Triangles or single branches represent EG species, which are indicated by their geographic origin and Arabic numbers. Species names in red are split into two species by the GMYC method (but not by the K/θ method). Species names in black are recognized by both methods. Numbers above branches are bootstrap values of 1000 replicates for NJ and ML trees (>50%), numbers below branches are posterior probabilities of Bayesian Inference.

Similar phylogenetic groupings were obtained regardless of the method used for tree construction (Neighbor joining, Maximum Likelihood or Bayesian Inference). The 24 sequences of *D*. *stevensoni* formed one large clade with 89–98% bootstrap support and a posterior probability of 0.99 (Fig. 1); this clade was further separated into three phylogenetic groups containing all European specimens but one in *Europe 1*, a second with the two specimens from South Africa (*Africa 1*) clustering together with one specimen from Spain (*Europe 2*), and a third clade with all American *D. stevensoni* with a split between specimens from South (*America 1*) and North (*America 2*) America.

The *Penthesilenula* species fell into 10 different clades: two clades of *Penthesilenula aotearoa* from Brazil, *P. kohanga*, *P. reidae*, five Brazilian clades, one European and one Australian clade of *P. brasiliensis*, respectively.

### Statistical Tests for EG Species Status

We applied the GMYC model [26] in four different ways (see Table 1): with the single or multiple threshold [52] model for shifts of branching rates and to the entire data set (without the outgroup) or a pruned data set only containing unique sequences of the ingroup, similarly to [53]. If all COI sequence data were used, the log likelihood (LL) of the ML tree with the GMYC model was significantly higher than the LL of the null model and this for both the single and multiple threshold models (Table 1). The multiple threshold model was significantly favored. While the single threshold model identified 13 species, the multiple threshold model identified 17 with a confidence interval (CI) of 16 to 17 (see Table 1 and Fig. 1).

**Table 1:** Summary of the analyses for the presence of darwinulid species with the GMYC method.

Data set	n	Single treshold				Multiple treshold			Single vs multiple treshold
		LL null model	LL GMYC	Number of species (CI)	LL null model		LL GMYC	Number of species(CI)	
COI, complete	58	400.59	561.59***	13 (13)	400.59		577.45***	17 (16–17)	**
COI, pruned	29	148.92	154.76**	1 (1)	148.92		151.62	5 (4–7)	n.s.
ITS2, complete	22	137.53	170.30***	4 (4)	137.53		169.32***	7 (7)	n.s.
ITS2, pruned	14	65.21	66.94	4 (1–5)	65.21		66.94	4 (1–7)	n.s.

n = number of sequences. LL = likelihood. CI = confidence interval. vs = versus. The complete data sets contain all sequences of the ingroup without the outgroup. The pruned data sets only contain unique sequences of the ingroup without the outgroup.

** p<0.01.

*** p<0.001. n.s.  =  not significant.

When applying the GMYC model to the pruned COI tree, the LL of the ML tree with the GMYC model was significantly higher for the single threshold model, but not for the multiple threshold model (Table 1). The latter was not statistically favored. Whereas the single threshold GMYC model identified a single EG species, the multiple threshold GMYC model recognized 5 species with a CI of 4 to 7.

The K/θ method recognized 16 species (see Fig. 1 and Table 2), thus one less than the GMYC model with the highest LL. Only the GMYC model splits *D. stevensoni Europe 1* further into two different species.

**Table 2:** Summary of results of K/θ tests for darwinulid species with the COI data set.

Clades tested	K	θ	K/θ	n1, n2	p(2 clades)
*P. aotearoa Brazil 1* & *Brazil 2* *	0.04439	0.00667	6.65	5, 2	>0.98
*P. brasiliensis Europe 1* &*Australia 1*	0.11017	0.00535	20.67	2, 2	>0.99
*P. brasiliensis Brazil 3* &*Brazil 4*	0.0557	0.00118	47.32	10, 3	>0.99
*P. brasiliensis Brazil 1* &*P. reidae*	0.13451	0.01073	12.53	2, 1	>0.99
*P. brasiliensis Brazil 5* &*P. kohanga*	0.13451	0.02178	6.17	2, 1	>0.98
*D. stevensoni Europe 2* &*Africa 1* **	0.02155	0.00533	4.04	2, 1	0.94
*D. stevensoni America 1* &*America 2*	0.05291	0.00269	19.7	3, 1	>0.98

Quantities K, θ, K/θ, n1, and n2 are defined in the text. p(2 clades) is the inferred probability that the 2 clades comprise samples from different clades in nature. All of the K/θ ratios are greater than 4, so the probability that the clades are samples from different evolutionary species is >0.95 in each case. The clades tested are sister clades in the first 2 and last 3 cases; in the other 3 cases, the two clades are not sister clades, but they have the lowest K values. A neighbor-joining tree was used to identify pairs of sister clades, some of which are different from those in the Bayesian tree in Figure 1.

* In this case we used the smaller value of *d* because the larger value is based on a sample size of 2 so that θ = dn/(n−1) = 2 d, which is likely to be an over-correction.

** The probability that the samples come from two different clades is 0.94 instead of 0.95 but K/θ >4 and the samples come from distant regions.

### ITS2

Following jModelTest, the best evolutionary model for all ITS2 sequences (with outgroup) was TPM2uf+G, with base frequencies of A = 0.13, C = 0.30, G = 0.33, T = 0.23 and rate matrices [A–C] = 2.36, [A–G] = 4.61, [A–T] = 2.36, [C–G] = 1.0, [C–T] = 4.61, [G–T] = 1.0 and a gamma distribution shape parameter of 0.45. Likelihoods of the ML trees constructed from ITS2 with and without clock assumption in PAUP did not differ significantly from each other (−2ΔLL = 13.922 df = 21, p>0.10), thus indicating that we could apply a universal molecular clock to construct the ultrametric tree (but see [51]).

### Phylogenetic Relationships

**Figure 2:**
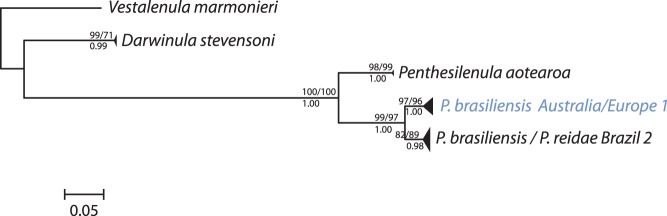
ITS phylogeny of the three darwinulid genera. This phylogeny was obtained by Bayesian Inference. Height of triangles reflects the number of sequences. Triangles or single branches represent EG species, which are indicated by their geographic origin and Arabic numbers. Species names in blue are only recognized by the K/θ method (but not by the GMYC method). Species names in black are recognized by both methods. Numbers above branches are bootstrap values of 1000 replicates for NJ and ML trees (>50%), numbers below branches are posterior probabilities of Bayesian Inference.

The ITS2 trees were similar in their topology, regardless which method was used for phylogenetic reconstructions. Six phylogenetic clades could be distinguished, mainly following the traditional morphospecies with good statistical support (Fig. 2): *Darwinula stevensoni*, *Penthesilena aotearoa*, and two clades with *P. brasiliensis*: one from Australia and Europe (*Australia/Europe 1*) and a second from Brazil, also including *P. reidae*.

### Statistical Tests for EG Species Status

As with the COI data, we applied the GMYC model to both, the entire ITS2 sequence data set without the outgroup and to the pruned data set only containing unique ITS2 sequences of the ingroup using a single or a multiple threshold model. If the ML tree from the complete data set was used, the LL of the GMYC model was significantly higher than the null model, regardless whether a single or a multiple threshold model was applied (see Table 1). The multiple threshold model was not statistically favored. The number of recognized species using the entire ITS2 data set varied between 4 with the single and 7 with the multiple threshold model (see Table 1 and Fig. 2). If the ML tree was constructed from the pruned ITS2 data set, the LL of the GMYC model was not significantly larger than the LL of the null model regardless which threshold model was used (Table 1). Both the single and multiple threshold model recognized four species (Fig. 2) with slightly different CI (Table 1).

Likewise, the K/θ method identified these four species plus an additional one (all with a probability >99%) splitting the *Australia/Europe 1* species of *Penthesilenula brasiliensis* further into two different species (Fig. 2), following their geographic distribution.

## Discussion

Both markers and both methods identified EG species in darwinulid ostracods morphospecies and also revealed cryptic diversity. Our results thus confirm the findings of others, namely that species are found in asexual organisms (see for example [17], [20]–[23], [25], [54]). Also the fact that we found cryptic species in darwinulid ostracods is not entirely surprising, given that cryptic diversity is common in metaozoan taxa [55] (but see [56]) and that more than 35 cryptic species were identified in a single ostracod morphospecies with mixed reproduction [28]. However, to have such a high level of cryptic diversity and genetic discontinuity in one of the major model groups of putative ancient asexuals demands attention.

### Using Different Methods to Detect EG Species

We applied the GMYC model in four different ways to each data set (see Table 1). The highest LL were obtained when including all sequences of the ingroup from both mitochondrial and nuclear sequence data, and a multiple threshold for the COI data. These conditions yielded estimates of species numbers being very close to the outcome of the K/θ method (see below). With the pruned data sets, the number of sequences was obviously too low for the GMYC model to detect the transition points. This is illustrated by the fact that the GMYC model was statistically not preferred over the null model for COI (multiple threshold model) and for ITS2 (both threshold models; see Table 1). The recognition of a single EG species (Table 1) by the GMYC model with the pruned COI data set does not make any biological sense as it contrasts sharply with the taxonomic evidence for five different morphospecies in our data. If we only consider the results of the GMYC with the highest LL, the number of EG species recognized by GMYC and K/θ is rather similar (see Fig. 1 & 2), namely 17 and 16 species, respectively, with COI, and five and four species, respectively, with ITS2. The differences between both methods are rather small and consist of further splitting of one well-supported species each from the same continent. In the COI tree, the GMYC method recognizes six *Darwinula stevensoni* species splitting *D. stevensoni Europe 1* further (Fig. 1) while the K/θ method identifies only five species in *D. stevensoni*. In the ITS2 tree, the K/θ method recognizes three species in *Penthesilenula brasiliensis* instead of two, following their continental distribution (Fig. 2). The two methods have different theoretical rationales and seem to be equally useful in testing whether species are present in asexuals and whether these also contain cryptic species. It is increasingly common practice in systematics to divide morphospecies into cryptic species when phylogenetic analyses show that the species consists of two or more well-supported clades. However, such clades can be formed by stochastic events within a single species. The K/θ and GMYC methods distinguish clades within a species from those that represent independently evolving populations, i.e. whole species. Our analyses of the two darwinulid genera, *Darwinula* and *Penthesilenula,* revealed well-supported clades to which EG species status was not attributed by either method (see Fig. S1) and thus clearly prove that both methods are powerful tools to quantitatively test for EG species.

### Incongruence of Nuclear and Mitochondrial Sequence Data

We have used COI and ITS2 sequence data to apply two methods of the EG species concept to two genera of putative ancient asexual ostracods. When comparing the mitochondrial patterns to those from the nuclear genome, we found that mitochondrial sequence data allowed for the detection of more (cryptic) species in these putative ancient asexuals than nuclear sequences, regardless of which of the two methods was used. With the COI data, seven cryptic EG species were identified in the genus *Penthesilenula* (five cryptic *P. brasiliensis* species, two *P. aotearoa* species plus *P. reidae*, and *P. kohanga*) and five to six in *Darwinula stevensoni*, but only four to five EG species were found with ITS2.

*Penthesilenula reidae* consistently clustered together with *P. brasiliensis* (see Fig.1) in our phylogenies but its species status was confirmed when both methods were applied to the COI data. This finding fits with the observed difference in valve morphology as compared to *P. brasiliensis* [37]. When using the ITS2 data, however, both methods grouped *P. reidae* together with *P. brasiliensis* as the same EG species, illustrating that the choice of molecular marker for species definitions in the Darwinulidae can affect congruence with morphological patterns.

Comparable studies using COI (mitochondrial) and EF1 alpha (nuclear) sequence data found congruent patterns between both marine (sexual) Bryozoa [57] and rotifers with mixed reproduction [58]. COI has been used successfully in numerous studies on species status [17]–[23], [25] and barcoding [59], [60], [61] (and others). Our observations on incongruence in the use of mitochondrial and nuclear data fit with previous results from darwinulids, indicating different mutation rates for the two markers [43], [46]. Thus, ribosomal regions are less likely to detect species, because of the lower substitution rates of these regions or their intrinsic properties [62].

### EG Species and Speciation in Darwinulid Ostracods

**Figure 3:**
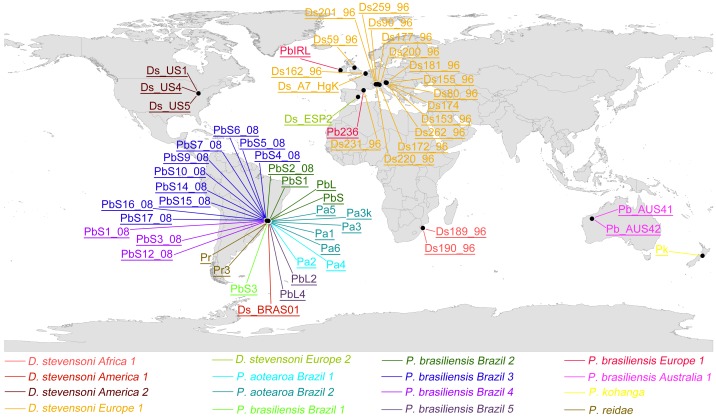
Global distribution of the EG species, determined by COI sequence data. Letters and numbers in the map refer to the analyzed specimens (see Table S1 for more details). Different EG species are indicated by different color codes.

**Figure 4:**
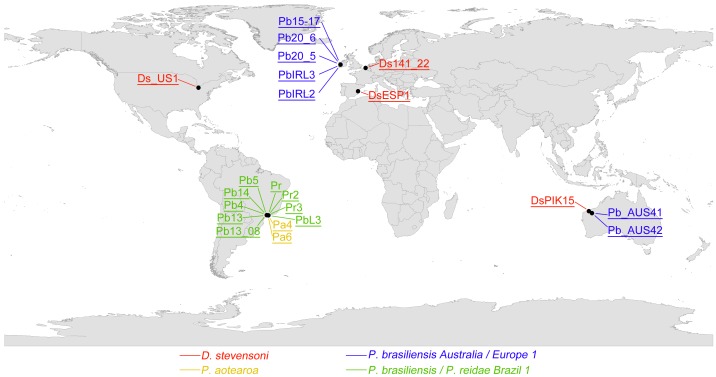
Global distribution of the EG species, determined by ITS2 sequence data. Letters and numbers in the map refer to the analyzed specimens (see Table S2 for more details). Different EG species are indicated by different color codes.

Since the EG species concept split both *Darwinula stevensoni* and *Penthesilenula brasiliensis* into different species, mainly according to continental distribution (Fig. 3 and Fig. 4), it seems that speciation processes might have been shaped by vicariance in these lineages, following continental drift. The cryptic species from Brazil and Australia in the *P. brasiliensis* complex, for example, may have become separated around 100–65 myr ago, when the South American continent split off Gondwana [63]. Such ancient vicariant processes, however, could not be the only ones leading to speciation in ancient asexual darwinulids, as shown by the lack of congruence between vicariant events and the split between European and Australian *P. brasiliensis* on the one hand and the phylogenetic polytomy of *D. stevensoni* on the other hand. Furthermore, two to three cryptic species of *D. stevensoni* are present within Europe and five cryptic species of *P. brasiliensis* and two cryptic species of *P. aotearoa* came from the same Brazilian province and some even from the same locality. Also different valve morphologies did not separate all cryptic *P. brasiliensis* species. While Brazilian *P. brasiliensis Brazil 3* and *4* indeed contained only small valve forms and *P. brasiliensis Brazil 5* only large forms, *P. brasiliensis Brazil 2* consisted of a mixture of both valve forms. It is also possible that the separation was caused by allopatric segregation at micro-scales, which is compatible with the small size and the limited dispersal abilities of darwinulid ostracods as compared to other freshwater ostracods [33], [64] and similar to what was described for the bdelloid rotifers *Philodina flaviceps* [22] and different *Adineta* species [25]. Another potential explanation could be that the cryptic *P. brasiliensis* and *P. aotearoa* species constituted different colonization events from genetically different and distant source populations. This hypothesis could be tested by investigating additional (Brazilian) samples more distant to our current study areas. Finally, finding of these cryptic species in close proximity could also be explained by different ecological requirements.

Such ecological differentiation has been described in bdelloid rotifers [16], [17] and oribatid mites [54], [65], [66]. Within the family Darwinulidae, no clear ecological specialization has so far been found. Darwinulid ostracods can occur in different water bodies and also in semi-terrestrial habitats [36]–[38] and ecological strategies within the family are rather diverse: some darwinulids either have a rather narrow salinity tolerance, e.g. *Vestalenula molopoensis* (Rossetti & Martens 1997) [67], or their salinity and temperature tolerance lies between that of a general purpose genotype (GPG) and a specialist [68], for example for *P. aotearoa* (Rossetti et al. 1998). On the other hand, *Darwinula* s*tevensoni* and *Penthesilenula brasiliensis* Pinto and Kotzian, 1961 both seem to have developed a GPG making them extremely tolerant of a wide range of temperatures and salinities [67], [68].

One may hypothesize that all cryptic species of the latter two morphospecies have a GPG, although evidence is currently lacking. In the light of our EG results, however, it might be worthwhile conducting additional experiments to verify whether these cryptic species vary in their ecology, and if thus ecological differentiation might have caused cryptic speciation. Divergent selection in feeding morphology of bdelloid rotifers has been observed [20]. That the same applies to putative ancient asexual darwinulid ostracods is less likely, given the high morphological uniformity within *Darwinula stevensoni* [45] and the low morphological differences between morphospecies [34]. Besides, additional biogeographic analyses of bdelloid rotifers [24] showed that there is only a low degree of habitat specialization in these microscopic organisms.

It is likely that more cryptic species in darwinulid ostracods will be identified when analyzing additional samples (see [69] and [70] on a discussion of the effects of incomplete sampling on the results of the GMYC method.) This will not change our initial result, namely that we have found multiple EG species within three darwinulid morphospecies, *Penthesilenula brasiliensis*, *P. aotearoa* and *Darwinula stevensoni*.

#### Comparison between the two darwinulid genera

If we compare the newly observed and mostly cryptic molecular diversity to the number of recent morphospecies known in the two genera, *Penthesilenula* and *Darwinula*, it appears that the genus with the largest number of recent morphospecies also comprises the highest cryptic diversity. This is of course in line with logical expectation, but it also strengthens the paradox of uneven diversity within the different darwinulid lineages. Within Darwinulidae, there are two speciose genera (*Penthesilenula* and *Vestalenula* [44]) and two less speciose ones (*Alicenula* with 3 morphospecies and the seemingly ubiquitous and cosmopolitan *Darwinula* [44], [45]), and the enigmatic monospecific and short range endemic *Isabenula* [34]. Such incongruence in speciosity between sister taxa is not unusual, but in putative ancient asexuals it requires additional attention. It was already foreshadowed [71] that maybe not all darwinulids have the same genetic adaptations to long-term asexuality, in which case *Darwinula stevensoni* with 25 myr of asexual reproduction might be best adapted to ancient asexuality (for example through highly efficient DNA repair [72]). The genera *Penthesilenula* and *Vestalenula* might have less robust mechanisms to counter asexual molecular diversification, although it is unlikely that this would be linked to rare males [40], [71]. In any case, darwinulids appear to constitute a relevant model group to assess relationships between morphological and molecular disparity on the one hand and reproductive mode on the other.

#### Cryptic darwinulid diversity compared to other ancient asexuals

Our findings imply that speciation patterns and processes of the darwinulid genera *Penthesilenula* and *Darwinula* are comparable to those of other putative ancient asexual animals. The patterns and processes of cryptic species and speciation are to some extend analogous to what has been described for ancient asexual oribatid mites [12], [18] and some bdelloid rotifers [17]–[23], [25]. Patterns are clearly different from rotifers with mixed reproduction where haplotypes were shared between continents [58]. Continental drift is one possible, but surely not the only, factor driving speciation in putative ancient asexual ostracods, whereas ecological speciation remains to be demonstrated (see above). Why darwinulids remain less speciose than bdelloids and oribatid mites, is still unclear. It is not owing to less taxonomic or sampling efforts as compared to the other two groups of putative ancient asexual animals [34]. From the extensive fossil record of ostracods, we know that the Darwinulidae were once far more speciose. In the Palaeozoic, the superfamily Darwinuloidea comprised more than 200 species, the majority of which were lost during the Permian-Triassic mass extinction (c 250 myr ago). The family Darwinulidae was the only one within this superfamily of which a limited number of species survived this mass extinction [73]. Some of these other, extinct, families might have comprised some sexual species in the Palaeozoic, but the Darwinulidae most likely did not [74]. This could suggest that sexual reproduction may not have been of importance for speciation in this ostracod family.

Lower rates of evolution in non-marine ostracods, as have been suggested from results on molecular analyses of mitochondrial COI and nuclear ITS1 [43], is another possibility for generally low speciosity. Why recent darwinulid ostracods are less speciose than the other putative ancient asexual animals remains a challenging question.

### Conclusions

By applying the EG species concept to the ostracod genera *Penthesilenula* and *Darwinula*, we found that mitochondrial sequence data detect more cryptic species in these putative ancient asexuals than nuclear sequences. Cryptic species were identified in both genera, but were more numerous in the more speciose genus *Penthesilenula*. Similar to another ancient asexual animal group, namely the oribatid mites, intercontinental distributions, and subsequent vicariance, were identified as a possible factor leading to speciation in the darwinulids, but also the opposite pattern, namely local coexistence, was observed, similar to certain ancient asexual bdelloid rotifers. Which other evolutionary forces lead to the formation of cryptic species in the putative ancient asexual ostracods remains to be discovered.

## Materials and Methods

### DNA Extractions

DNA was extracted from individual ostracods, fixed in pure EtOH after washing the specimens in distilled water thrice and once in PBS prior to DNA extraction. Living specimens were left several hours in distilled water to expel the gut content before their DNA was extracted with the Qiagen DNA Easy Blood and Tissue kit, following the manufacture’s protocol. An overview on the analyzed specimens is provided in the supplementary material (Table S1 for COI and Table S2 for ITS2).

### PCR Amplification and Sequencing

Part of the mitochondrial COI gene was amplified with the universal primers HCO and LCO [75] and specific primers for *Penthesilenula* (LCO_Pb_Forward 5′-CTAAGAGTCTTAATTCGATCCGAAT & HCO_Pb_Reverse 5′-GGTATTCGGTCTATGGGTATTCC) and *Darwinula stevensoni* [46]. Obtained sequences resemble base pairs 93 to 477 and amino acids 31 to 129 of the Folmer fragment [75]. All PCR amplifications were conducted in 25 µl volumes with the Hot Star Master Mix from Qiagen (1.5 mM MgCl2, 0.1 µm primer, 200 µM dNTP, Tris·Cl, KCl, (NH_4_)_2_SO_4_, 1.25 U Taq ) and the following conditions: 15 min at 95°C, 40 cycles with 1 min at 95°C, 1 min at 40°C [75] or 45°C (specific primers), 1 min at 72°C and a final extension step for 10 min at 72°C in a T personal Thermoblock (Biometra).

ITS1 and ITS2 were first amplified with universal primers ITS1 & ITS4 [76] until specific primers PbITS2F (5′-AACGCGCTCTCTGGGGTTTTCCTCC) and PbITS2R (5′-TGGCGCCCTGCAATTCGCCGTGTT) could be developed for *Penthesilenula*. PCR amplifications were conducted as described above, except that annealing temperature was 50°C. From most specimens, ITS2 was sequenced directly (see below). For a few *P. brasiliensis* and *D. stevensoni* specimens, amplicons were cloned prior to sequencing with M13 primers as described in [47].

PCR products were checked by agarose gel electrophoresis with subsequent Sybersafe staining and gels were photographed. PCR products were cleaned with the GFX™ PCR DNA and gel band purification kit (GE Healthcare) according to the manufacture’s protocol and bi-directionally sequenced using the PCR primers and the Big Dye 3.1 kit (Applied Biosystems). Sequences were resolved on an ABI 3130×.

### Analyses of Sequence Data

Chromatograms were visualized with Chromas, both strands aligned with ClustalX [77] and ambiguities checked manually. Sequence identity was confirmed by BLAST [78] and for COI, by translating DNA sequence data into amino acids with MacClade [79]. Individual sequences were assembled and used for phylogenetic reconstructions. All sequences have been submitted to Genbank (accession numbers JX069208-JX069267; see Tables S1 and S2 for more details).

### Phylogenetic Reconstructions

We identified the model that best fitted our data with jModeltest [49] using 24 or 88 models. Phylogenetic trees were constructed with three different methods: NJ searches in PAUP [50], Bayesian Inference with MrBayes 3.1.2 [80]; with 4 million generations sampling every 100^th^ generation, a burn-in of 25% and using the parameters identified by jModeltest from 24 models and PhyML [81] for constructing Maximum Likelihood trees with bootstraps of 1000 replicates for each data set and applying the parameters of jModeltest for all 88 models.

### Application of Species Criteria

#### Using K/θ

Identifying evolutionary species using the K/θ ratio is a four-step procedure [17]. First, we used PAUP [50] to make neighbor-joining trees with an evolutionary model selected by jModelTest and identified clades being supported by at least 90% bootstraps. Second, we estimated the mean pairwise uncorrected sequence difference d for each clade and its sister clade or the closest phylogenetic clade and then the nucleotide diversity π = dn/(n−1) where n is the number of sequences in the clade. When all sequences in a clade were identical, we calculated d as if one sequence differed from each of the others at one site, in which case d = 2/Ln where L is the sequence length. The parameter θ ≈ 2 N_e_π where N_e_ is the effective population size and π is the mutation rate, is then estimated by θ = π/(1–4 π/3). Third, we calculated the mean pairwise sequence difference K between the pair of sister or closely related clades, using the evolutionary model selected by ModelTest to correct for multiple hits (GTR+I+G for COI, GTR+G for ITS2). Fourth, the ratio K/θ was calculated for each clade and used together with the sample sizes of the clade and its sister clade to enter a lookup table provided by Noah Rosenberg (personal communication) to find the probability that the two clades are samples from populations that have been evolving independently long enough to become reciprocally monophyletic (see [82] for the theory used to calculate the values). Note that in this procedure reciprocal monophyly is used as evidence of independent evolution and not as a species criterion by itself. Finally, clades that passed this test *and* have K/θ ≥4 are considered to be different species with probability ≥0.95.

#### Applying the GMYC model

We used the GMYC model of [26] to attribute species boundaries in *Penthesilenula* and *Darwinula* when applying the EG species concept. In order to construct the required ultrametic trees from COI and ITS2 sequence data without outgroups, we applied the parameters identified with jModeltest [49] to construct ML trees in PAUP [50], while enforcing a molecular clock and using heuristic tree searches. By repeating the ML analyses without a molecular clock and subsequently conducting a likelihood ratio test, we tested whether the assumption of equal rates amongst the branches of the tree with clock was not violated [but see 51]. The ultrametric trees were then used for the splits package by Barraclough et al. (https://r-forge.r-project.org/projects/splits/) implemented in R *version 2.12.2* [83] with the APE [84] and Geiger [85] libraries. Assuming constant speciation rates without extinction and neutral coalescence within each species, the GMYC model predicts branching rates at the species boundary by classifying the observed intervals of branching time either as being interspecific (diversification) or intraspecific (coalescent) processes. The split package estimates the LL with the GMYC model and without it (null model) and compares the LL in likelihood ratio tests. Also, the number of species-like clusters is provided together with the CI corresponding to the threshold values ±2 LL around the ML estimate (see equation 6 of [26]). The transition point between diversification and coalescent processes can either consist of a single or multiple tresholds, the latter permitting different node ages for the transition of branching rates [52]. Chi Square tests allow testing which threshold model is statistically favored.

## Supporting Information

Figure S1 **Details of part of the COI phylogeny.** The well-supported subclades within the EG species *P. brasiliensis Brazil 2* & *3* did not receive species status by the GMYC or the K/θ method.
(EPS)

Table S1 **Overview of specimens analysed for COI.**
(DOC)

Table S2 **Overview of specimens analysed for ITS2.**
(DOCX)
