# Supplementary material for: Cryptic Species in Putative Ancient Asexual Darwinulids (Crustacea, Ostracoda)
Source: PLoS One. 2012 Jul 3;7(7):e39844. doi: 10.1371/journal.pone.0039844 (PMC3389007; doi:10.1371/journal.pone.0039844)
Supplement: Table S1 — Overview of specimens analysed for COI. (DOC) [file pone.0039844.s002.doc]

# Table S1: Overview of specimens analysed for COI.

| **Species** | **Abbrev.** | **Morph** | **Locality** | **Coordinates** | **Country/**  **continent** | **Genbank Nr.** | **EG species** |
| --- | --- | --- | --- | --- | --- | --- | --- |
| *P. aotearoa* | Pa1 |  | Boracéia Biological Station, São Paulo State | 23°38’17”S  45°50’25”W | Brazil/  S America | JX069233 | *P. aotearoa Brazil 2* |
| *P. aotearoa* | Pa2 |  | Boracéia Biological Station, São Paulo State | 23°38’17”S  45°50’25”W | Brazil/  S America | JX069230 | *P. aotearoa Brazil 1* |
| *P. aotearoa* | Pa3 |  | Boracéia Biological Station, São Paulo State | 23°38’17”S  45°50’25”W | Brazil/  S America | JX069235 | *P. aotearoa Brazil 2* |
| *P. aotearoa* | Pa3k |  | Boracéia Biological Station, São Paulo State | 23°38’17”S  45°50’25”W | Brazil/  S America | JX069236 | *P. aotearoa Brazil 1* |
| *P. aotearoa* | Pa4 |  | Boracéia Biological Station, São Paulo State | 23°38’17”S  45°50’25”W | Brazil/  S America | JX069231 | *P. aotearoa Brazil 1* |
| *P. aotearoa* | Pa5 |  | Boracéia Biological Station, São Paulo State | 23°38’17”S  45°50’25”W | Brazil/  S America | JX069234 | *P. aotearoa Brazil 2* |
| *P. aotearoa* | Pa6 |  | Boracéia Biological Station, São Paulo State | 23°38’17”S  45°50’25”W | Brazil/  S America | JX069232 | *P. aotearoa Brazil 2* |
| *P. brasiliensis* | PbL | Large, elongated | Boracéia Biological Station, São Paulo State | 23°38’17”S  45°50’25”W | Brazil/  S America | JX069240 | *P. brasiliensis Brazil 2* |
| *P. brasiliensis* | PbL2 | Large, elongated | Boracéia Biological Station, São Paulo State | 23°38’17”S  45°50’25”W | Brazil/  S America | JX069241 | *P. brasiliensis Brazil 5* |
| *P. brasiliensis* | PbL4 | Large, elongated | Boracéia Biological Station, São Paulo State | 23°38’17”S  45°50’25”W | Brazil/  S America | JX069242 | *P. brasiliensis Brazil 5* |
| *P. brasiliensis* | PbS | Small, rectangular | Boracéia Biological Station, São Paulo State | 23°38’17”S  45°50’25”W | Brazil/  S America | JX069243 | *P. brasiliensis Brazil 2* |
| *P. brasiliensis* | PbS1 | Small, rectangular | Boracéia Biological Station, São Paulo State | 23°38’17”S  45°50’25”W | Brazil/  S America | JX069244 | *P. brasiliensis Brazil 2* |
| *P. brasiliensis* | PbS3 | Small, rectangular | Boracéia Biological Station, São Paulo State | 23°38’17”S  45°50’25”W | Brazil/  S America | JX069245 | *P. brasiliensis Brazil 1* |
| *P. brasiliensis* | PbS1_08 | Small, rectangular | Campus of the University of São Paulo | 23°33’51’’S  46°43’48’’W | Brazil/  S America | JX069246 | *P. brasiliensis Brazil 4* |
| *P. brasiliensis* | PbS2_08 | Small, rectangular | Campus of the University of São Paulo | 23°33’51’’S  46°43’48’’W | Brazil/  S America | JX069247 | *P. brasiliensis Brazil 2* |
| *P. brasiliensis* | PbS3_08 | Small, rectangular | Campus of the University of São Paulo | 23°33’51’’S  46°43’48’’W | Brazil/  S America | JX069248 | *P. brasiliensis Brazil 4* |
| *P. brasiliensis* | PbS4_08 | Small, rectangular | Campus of the University of São Paulo | 23°33’51’’S  46°43’48’’W | Brazil/  S America | JX069249 | *P. brasiliensis Brazil 3* |
| *P. brasiliensis* | PbS5_08 | Small, rectangular | Campus of the University of São Paulo | 23°33’51’’S  46°43’48’’W | Brazil/  S America | JX069250 | *P. brasiliensis Brazil 3* |
| *P. brasiliensis* | PbS6_08 | Small, rectangular | Campus of the University of São Paulo | 23°33’51’’S  46°43’48’’W | Brazil/  S America | JX069251 | *P. brasiliensis Brazil 3* |
| *P. brasiliensis* | PbS7_08 | Small, rectangular | Campus of the University of São Paulo | 23°33’51’’S  46°43’48’’W | Brazil/  S America | JX069252 | *P. brasiliensis Brazil 3* |
| *P. brasiliensis* | PbS9_08 | Small, rectangular | Campus of the University of São Paulo | 23°33’51’’S  46°43’48’’W | Brazil/  S America | JX069253 | *P. brasiliensis Brazil 3* |
| *P. brasiliensis* | PbS10_08 | Small, rectangular | Campus of the University of São Paulo | 23°33’51’’S  46°43’48’’W | Brazil/  S America | JX069254 | *P. brasiliensis Brazil 3* |
| *P. brasiliensis* | PbS12_08 | Small, rectangular | Campus of the University of São Paulo | 23°33’51’’S  46°43’48’’W | Brazil/  S America | JX069259 | *P. brasiliensis Brazil 4* |
| *P. brasiliensis* | PbS14_08 | Small, rectangular | Campus of the University of São Paulo | 23°33’51’’S  46°43’48’’W | Brazil/  S America | JX069255 | *P. brasiliensis Brazil 3* |
| *P. brasiliensis* | PbS15_08 | Small, rectangular | Campus of the University of São Paulo | 23°33’51’’S  46°43’48’’W | Brazil/  S America | JX069258 | *P. brasiliensis Brazil 3* |
| *P. brasiliensis* | PbS16_08 | Small, rectangular | Campus of the University of São Paulo | 23°33’51’’S  46°43’48’’W | Brazil/  S America | JX069257 | *P. brasiliensis Brazil 3* |
| *P. brasiliensis* | PbS17_08 | Small, rectangular | Campus of the University of São Paulo | 23°33’51’’S  46°43’48’’W | Brazil/  S America | JX069256 | *P. brasiliensis Brazil 3* |
| *P. brasiliensis* | Pb236 | Large, rectangular | Clue de la Fou | 42°48’03”N  02°29’56”E | France/  Europe | AF031303 | *P. brasiliensis Europe 1* |
| *P. brasiliensis* | PbIRL | Large, rectangular | Lough Lickeen | 52°57'46”N 09°13'46”W | Ireland/  Europe | AJ319738 | *P. brasiliensis Europe 1* |
| *P. brasiliensis* | Pb_AUS41 | Large, rectangular | Circular Pool Springs,  Dales Gorge, Karijini National Park | 22°28’36”S 118°33’37”E | Australia/  Australia | JX069238 | *P. brasiliensis, Australia 1* |
| *P. brasiliensis* | Pb_AUS42 | Large, rectangular | Circular Pool Springs,  Dales Gorge, Karijini National Park | 22°28’36”S 118°33’37”E | Australia/  Australia | JX069239 | *P. brasiliensis, Australia 1* |
| *P. reidae* | Pr |  | Boracéia Biological Station, São Paulo State | 23°40’01”S  45°54’01”W | Brazil/  S America | JX069260 | *P. reidae* |
| *P. reidae* | Pr3 |  | Boracéia Biological Station, São Paulo State | 23°40’01”S  45°54’0.1”W | Brazil/  S America | JX069261 | *P. reidae* |
| *P. kohanga* | Pk |  | Kinnoull Station, Makara, Wellington, New Zealand | 41°19'S  174°41'E | New Zealand/  Australia | JX069237 | *P. kohanga* |
| *D. stevensoni* | Ds155_96 |  | Keutschacher See | 46°35’17”N  14°09’12”E | Austria/  Europe | AF031297 | *D. stevensoni Europe 1* |
| *D. stevensoni* | Ds80_96 |  | Wörther See | 46°38’01”N  14°08’25”E | Austria/  Europe | AF031295 | *D. stevensoni Europe 1* |
| *D. stevensoni* | Ds200_96 |  | Lago di Garda | 45°32’14”N  10°33’30”E | Italy/  Europe | AF031294 | *D. stevensoni Europe 1* |
| *D. stevensoni* | Ds177_96 |  | Montorfarno | 45°47’11”N  09°08’46”E | Italy/  Europe | AF031293 | *D. stevensoni Europe 1* |
| *D. stevensoni* | Ds231_96 |  | Clue de la Fou | 42°48’03”N  02° 29’56”E | France/  Europe | AF031302 | *D. stevensoni Europe 1* |
| *D. stevensoni* | Ds220_96 |  | Annone | 45°48’12”N  09°20’14”E | Italy/  Europe | AF031301 | *D. stevensoni Europe 1* |
| *D. stevensoni* | Ds181_96 |  | Afritzer See | 46°44’25”N  13°46’20”E | Austria/  Europe | AF031300 | *D. stevensoni Europe 1* |
| *D. stevensoni* | Ds174 |  | Ossiacher See | 46°40’36”N  13°58’51”E | Austria/  Europe | AF031299 | *D. stevensoni Europe 1* |
| *D. stevensoni* | Ds153_96 |  | Faaker See | 46°34’59”N  13°55’07”E | Austria/  Europe | AF031296 | *D. stevensoni Europe 1* |
| *D. stevensoni* | Ds262_96 |  | Keutschacher See | 46°35’17”N  14°09’12” E | Austria/  Europe | AF031298 | *D. stevensoni Europe 1* |
| *D. stevensoni* | Ds90_96 |  | Lago di Monate | 45°48’13”N  08°39’53”E | Italy/  Europe | AF031289 | *D. stevensoni Europe 1* |
| *D. stevensoni* | Ds259_96 |  | Lago di Monate | 45°48’13”N  08°39’53”E | Italy/  Europe | AF031288 | *D. stevensoni Europe 1* |
| *D. stevensoni* | Ds_A7_HgK |  | Hollandersgaatkreek | 51°16’08”N  03°32’07”E | Belgium/  Europe | JX069262 | *D. stevensoni Europe 1* |
| *D. stevensoni* | Ds59_96 |  | Semerwater | 54°16’37”N  02°07’28”E | UK/  Europe | AF031292 | *D. stevensoni Europe 1* |
| *D. stevensoni* | Ds162_96 |  | Blikkreek | 51°17’58”N  03°27’54”E | Belgium/  Europe | AF031290 | *D. stevensoni Europe 1* |
| *D. stevensoni* | Ds172_96 |  | Lago di Endine | 45°46’41”N  09°56’35”E | Italy/  Europe | AF031287 | *D. stevensoni Europe 1* |
| *D. stevensoni* | Ds201_96 |  | Comabbio | 45°46’14”N  08°41’60”E | Italy/  Europe | AF03129 | *D. stevensoni Europe 1* |
| *D. stevensoni* | Ds190_96 |  | Lake Sibaya | 27°18’35”S  32°34’24”E | South Africa/  Africa | AF031286 | *D. stevensoni Africa 1* |
| *D. stevensoni* | Ds189_96 |  | Lake Sibaya | 27°18’35”S  32°34’24”E | South Africa/  Africa | AF031286 | *D. stevensoni Africa 1* |
| *D. stevensoni* | Ds_ESP2 |  | La Albufera, Valencia | 39°20’53”N  00°19’27”W | Spain/  Europe | JX069263 | *D. stevensoni Europe 2* |
| *D. stevensoni* | Ds_US4 |  | Herrick Fen Nature Preserve, Ohio | 41°12’50”N  81°22’16”W | USA/  N America | JX069264 | *D. stevensoni America 2* |
| *D. stevensoni* | Ds_US5 |  | Herrick Fen Nature Preserve, Ohio | 41°12’50”N  81°22’16”W | USA/  N America | JX069265 | *D. stevensoni America 2* |
| *D. stevensoni* | Ds_US1 |  | Herrick Fen Nature Preserve, Ohio | 41°12’50”N  81°22’16”W | USA/  N America | JX069266 | *D. stevensoni America 2* |
| *D. stevensoni* | Ds_BRAS01 |  | Boracéia Biological Station, São Paulo State | 23°38’17”S  45°50’25”W | Brazil/  S America | JX069267 | *D. stevensoni America 1* |
| *V. molopoensis* | Vmol |  |  |  | South Africa/  Africa | [AJ534411](../../../entrez/viewer.fcgi%3Fdb=nuccore&id=30519667) | Outgroup |

Abbr. = abbreviation. Differences in valve morphs were only found in the morphospecies *Penthesilenula brasiliensis*, not in the other analysed morphospecies. P = *Penthesilenula*. D = *Darwinula*. V = *Vestalenula*. S = South. N = North. The GMYC method splits *D. stevensoni Europe 1* further into two different species.
