# Supplementary material for: Cryptic Species in Putative Ancient Asexual Darwinulids (Crustacea, Ostracoda)
Source: PLoS One. 2012 Jul 3;7(7):e39844. doi: 10.1371/journal.pone.0039844 (PMC3389007; doi:10.1371/journal.pone.0039844)
Supplement: Table S2 — Overview of specimens analysed for ITS2. (DOCX) [file pone.0039844.s003.docx]

**Table S2: Overview of specimens analysed for ITS2.**

| **Species** | **Abbr.** | **Morph** | **Locality** | **Coordinates** | **Country/**  **continent** | **Genbank Nr.** | **EG species** |  |
| --- | --- | --- | --- | --- | --- | --- | --- | --- |
| *P. brasiliensis* | PbL3 | Small, elongated | Boracéia Biological Station, São Paulo State | 23°38’17”S  45°50’25”W | Brazil/  S America | JX069218 | *P. brasiliensis/P. reidae Brazil 1* |  |
| *P. brasiliensis* | Pb5 | Small, elongated | Campus of the University of São Paulo | 23°33’51’’S  46°43’48’’W | Brazil/  S America | JX069217 | *P. brasiliensis/P. reidae Brazil 1* |  |
| *P. brasiliensis* | Pb14 | Small, elongated | Campus of the University of São Paulo | 23°33’51’’S  46°43’48’’W | Brazil/  S America | JX069216 | *P. brasiliensis/P. reidae Brazil 1* |  |
| *P. brasiliensis* | Pb4 | Small, elongated | Campus of the University of São Paulo | 23°33’51’’S  46°43’48’’W | Brazil/  S America | JX069213 | *P. brasiliensis/P. reidae Brazil 1* |  |
| *P. brasiliensis* | Pb13 | Small, elongated | Campus of the University of São Paulo | 23°33’51’’S  46°43’48’’W | Brazil/  S America | JX069215 | *P. brasiliensis/P. reidae Brazil 1* |  |
| *P. brasiliensis* | Pb13_08 | Small, elongated | Campus of the University of São Paulo | 23°33’51’’S  46°43’48’’W | Brazil/  S America | JX069214 | *P. brasiliensis/P. reidae Brazil 1* |  |
| *P. brasiliensis* | Pb_IRL2 | Small, elongated | Lough Lickeen | 52°57'46”N 09°13'46”W | Ireland/  Europe | JX069221 | *P. brasiliensis/P. reidae Brazil 1* |  |
| *P. brasiliensis* | Pb20_5 | Small, elongated | Lough Lickeen | 52°57'46”N 09°13'46”W | Ireland/  Europe | JX069222 | *P. brasiliensis Australia/Europe 1* |  |
| *P. brasiliensis* | Pb20_6 | Small, elongated | Lough Lickeen | 52°57'46”N 09°13'46”W | Ireland/  Europe | JX069223 | *P. brasiliensis Australia/Europe 1* |  |
| *P. brasiliensis* | Pb_IRL3 | Small, elongated | Lough Lickeen | 52°57'46”N 09°13'46”W | Ireland/  Europe | JX069220 | *P. brasiliensis Australia/Europe 1* |  |
| *P. brasiliensis* | Pb_15 | Small, elongated | Lough Lickeen | 52°57'46”N 09°13'46”W | Ireland/  Europe | JX069219 | *P. brasiliensis Australia/Europe 1* |  |
| *P. brasiliensis* | Pb_AUS41 | Large, rectangular | Circular Pool Springs,  Dales Gorge, Karijini National Park | 22°28’36’’S 118°33’37’’E | Australia/  Australia | JX069224 | *P. brasiliensis Australia/Europe 1* |  |
| *P. brasiliensis* | Pb_AUS42 | Large, rectangular | Circular Pool Springs,  Dales Gorge, Karijini National Park | 22°28’36’’S 118°33’37’’E | Australia/  Australia | JX069225 | *P. brasiliensis Australia/Europe 1* |  |
| *P. reidae* | Pr |  | Boracéia Biological Station, São Paulo State | 23°40’00.7”S  45°54’0.89”W | Brazil/  S America | JX069210 | *P. brasiliensis/P. reidae Brazil 1* |  |
| *P. reidae* | Pr2 |  | Boracéia Biological Station, São Paulo State | 23°40’00.7”S  45°54’0.89”W | Brazil/  S America | JX069212 | *P. brasiliensis/P. reidae Brazil 1* |  |
| *P. reidae* | Pr3 |  | Boracéia Biological Station, São Paulo State | 23°40’00.7”S  45°54’0.89”W | Brazil/  S America | JX069211 | *P. brasiliensis/P. reidae Brazil 1* |  |
| *P. aotearoa* | Pa6 |  | Boracéia Biological Station, São Paulo State | 23°38’17”S  45°50’25”W | Brazil/  S America | JX069209 | *Penthesilenula aotearoa* |  |
| *P. aotearoa* | Pa4 |  | Boracéia Biological Station, São Paulo State | 23°38’17”S  45°50’25”W | Brazil/  S America | JX069208 | *Penthesilenula aotearoa* |  |
| *D. stevensoni* | Ds141_22 |  | Hollandersgaatkreek | 51°16’08”N  03°32’07”E | Belgium/  Europe | AJ534960 | *Darwinula stevensoni* |  |
| *D. stevensoni* | DsPIK15 |  | Gregory Gorge, WA | 21°32”59’S 116°58”15’E | Australia/  Australia | JX069226 | *Darwinula stevensoni* |  |
| *D. stevensoni* | DsESP1 |  | La Albufera, Valencia | 39°20’53”N  00°19’27”W | Spain/  Europe | JX069228 | *Darwinula stevensoni* |  |
| *D. stevensoni* | Ds_US1 |  | Herrick Fen Nature Preserve, Ohio | 41°12’50”N  81°22’16”W | USA/  N America | JX069227 | *Darwinula stevensoni* |  |
| *V. marmonieri* | Vmar |  |  |  | Australia | JX069229 | outgroup |  |

Abbr. = abbreviation. Differences in valve morphs were only found in the morphospecies *Penthesilenula brasiliensis*, not in the other analysed morphospecies. P = *Penthesilenula*. D = *Darwinula*. V = *Vestalenula*. S = South. N = North. The K/θ method splits the EG species *P. brasiliensis Australia/Europe 1* further into two different species following geographic distribution.
